# Pipeline versus Tubridge in the treatment of unruptured posterior circulation aneurysms

**DOI:** 10.1186/s41016-023-00337-0

**Published:** 2023-08-04

**Authors:** Hengwei Jin, Jian Lv, Xiangyu Meng, Xinke Liu, Hongwei He, Youxiang Li

**Affiliations:** 1 Department of Neurosurgery, Beijing Tiantan Hospital and Beijing Neurosurgical Institute, Capital Medical University, No.119, South 4Th Ring West Road, Fengtai District, Beijing, 100070 China; 2 Neurosurgery Department, The First Hospital of Hebei Medical University, Donggang Road 89, Shijiazhuang, Hebei Province China

**Keywords:** Pipeline embolization device, Tubridge flow diverter, Posterior circulation aneurysm

## Abstract

**Background:**

To compare the safety and efficacy of pipeline embolization device (PED) and Tubridge flow diverter (TFD) for unruptured posterior circulation aneurysms.

**Methods:**

Posterior aneurysm patients treated with PED or TFD between January, 2019, and December, 2021, were retrospectively reviewed. Patients’ demographics, aneurysm characteristics, treatment details, complications, and follow-up information were collected. The procedural-related complications and angiographic and clinical outcome were compared.

**Results:**

A total of 107 patients were involved; PED was applied for 55 patients and TFD for 52 patients. A total of 9 (8.4%) procedural-related complications occurred, including 4 (7.3%) in PED group and 5 (9.6%) in TFD group. During a mean of 10.3-month angiographic follow-up for 81 patients, complete occlusion was achieved in 35 (85.4%) patients in PED group and 30 (75.0%) in TFD group. The occlusion rate of PED group is slightly higher than that of TFD group. A mean of 25.0-month clinical follow-up for 107 patients showed that favorable clinical outcome was achieved in 53 (96.4%) patients in PED group and 50 (96.2%) patients in TFD group, respectively. No statistical difference was found in terms of procedural-related complications (*p* = 0.737), occlusion rate (*p* = 0.241), and favorable clinical outcome (0.954) between groups.

**Conclusions:**

The current study found no difference in complication, occlusion, and clinical outcome between PED and TFD for unruptured PCAs.

## Background

Posterior circulation aneurysms (PCAs) comprise 10–15% of all cerebral aneurysms [1]. Treatment of PCA is still challenging either by endovascular or surgical strategies due to the complexity of these lesions [2]. Overall, endovascular methods have yielded slightly better results than microsurgery and were considered to be the primary treatment modality [3]. Flow diversion device (FD) is a common endovascular treatment for PCAs. The same mechanism of action applies to all flow diverters, but devices differ in their materials and designs [4]. The pipeline embolization device (PED, Medtronic, USA) was the first FD approved for clinical use and is currently the most reliable FD with respect to clinical and laboratory evidence [5–7]. Tubridge flow diverter (TFD; MicroPort, China) is a relatively new type of flow diverter, made with nickel-titanium-braided microfilament [8]. In recent years, sporadic studies have compared the safety and efficacy of different flow diverter treatments for intracranial aneurysms [4, 9, 10], while none has compared the safety and efficacy of PED and TFD for unruptured PCAs. In the current study, we make a retrospective study comparing the safety and clinical efficacy of the PED and TFD for PCAs. This study would provide a reference for FD selection in patients with various PCAs.

## Methods

### Patients

All patients signed informed consent. Patients with PCA treated with PED or TFD between January, 2019, and December, 2021, were retrospectively reviewed. Patients with ruptured PCAs were excluded. Patients who refuse to participate or lost to follow-up were also excluded. Information includes the following: patient demographics, aneurysm characteristics, procedure details, complications, angiographic, and clinical outcome were collected.

### Endovascular procedure

Treatment decisions were made by consensus of multidisciplinary discussion including neurosurgeons, interventional neuroradiologists, and neurologists. Indications for using FDs include aneurysms with a high technical difficulty, high risk of recurrence with conventional endovascular or surgical methods, fusiform, dissecting, and large aneurysms. In order to decrease rupture risk and increase occlusion rate, adjacent coils were used for aneurysm with irregular shape (such as daughter aneurysm) and big or giant in size to promote intra-lumen thrombosis. All patients were pretreated with daily dual antiplatelet drugs consisting of 75-mg clopidogrel and 100-mg aspirin for at least 5 days before the procedure. All patients received platelet inhibition rate and CYP2C19 gene-type testing. For patients with unsatisfactory platelet inhibition rate or clopidogrel resistance gene type (intermediate metabolic or poor metabolic type), ticagrelor is a candidate therapy. Dual antiplatelet therapy is prescribed for 6 months, and aspirin alone is prescribed for 12 months after the procedure.

Endovascular treatment (EVT) was performed under general anesthesia through transfemoral arterial access. A 6- to 8-F sheath was inserted through the femoral artery, and guiding catheter was navigated into dominant vertebral artery. The PCA was embolized with FD according to standardized routine of our institution. The FDs included the PED and the TFD. Patients received a bolus of 3000 to 5000 IU heparin just before the deployment of stent, followed by 1000 IU/h heparin to maintain the activated clotting time above 250 s.

### Complications and follow-up

Before and immediately after the procedure, the neurological function of every patient was evaluated. The mRS at presentation, immediate after procedure, and at discharge were recorded. Procedural-related complication is defined as any additional neurological deficit compared with pre-operation and hemorrhage or infarction confirm by CT/MRI. Angiographic and clinical follow-up were performed for all patients. Angiographic follow-up was based on DSA or CTA performed ≥ 6 months after procedure. The angiographic results were categorized as complete and incomplete occlusion. The clinical outcome was evaluated according to modified Rankin Scale (mRS). Favorable clinical outcome was defined as mRS scores of 0–2, and unfavorable clinical outcome was defined as an mRS score of 3–6 at clinical follow-up.

### Statistical analysis

Categorical variables were presented as numbers and percentages, and continuous data were expressed using the mean ± standard deviation values. The chi-square and Fisher exact tests were used to compare categorical variables. Student’s *t*-test and Mann–Whitney *U*-test were used to compare continuously. Univariate and multivariate logistic analysis were performed to find factors associated with complications, occlusion, and unfavorable clinical outcomes. Differences with *p* < 0.05 were considered statistically significant. Data analyses were carried out using SPSS software (SPSS 22.0).

## Results

A total of 107 patients were involved, including 80 (74.8%) males and 27 (25.2%) females. Age ranges from 29 to 82 years old (mean 55.2 ± 10.0 years old). Clinical presentations include headache or dizziness (*n* = 48, 44.9%) and ischemic symptom (*n* = 7, 6.5%). Fifty-two (48.6%) patients have no symptom, and the aneurysm is incidentally found. A total of 69 (64.5%) patients had concomitant hypertension, and 11 (10.3%) patients had concomitant diabetes, respectively. The initial mRS is 0 in 103 patients and 1 in 4 patients (Table 1).

**Table 1:** Demographics and aneurysm characteristics of patients

	Total *n* = 107	PED *n* = 55	TFD *n* = 52	*P*-value
Gender (*n*, %)				0.696
Male	80 (74.8)	42 (76.4)	38 (73.1)	
Female	27 (25.2)	13 (23.6)	14 (26.9)	
Age (mean ± SD)	55.2 ± 10.0	53.4 ± 9.6	57.0 ± 10.2	0.063
Clinical presentation (n, %)				0.117
Headache or dizziness	48 (44.9)	30 (54.5)	18 (34.6)	
Ischemic symptom	7 (6.5)	3 (5.5)	4 (7.7)	
Incidentally found	52 (48.6)	22 (40.0)	30 (57.7)	
Hypertension (*n*, %)	69 (64.5)	33 (60.0)	36 (69.2)	0.319
Diabetes (*n*, %)	11 (10.3)	5 (9.1)	6 (11.5)	0.677
Multiple aneurysms (*n*, %)	8 (7.5)	6 (10.9)	2 (3.8)	0.165
Location (*n*, %)				0.999
Vertebral artery	83 (77.6)	43 (78.2)	40 (76.9)	
Basilar artery	16 (15.0)	8 (14.5)	8 (15.4)	
Vertebrobasilar artery	6 (5.6)	3 (5.5)	3 (5.8)	
Others	2 (1.9)	1 (1.8)	1 (1.9)	
Morphology (*n*, %)				0.281
Saccular	32 (29.9)	19 (34.5)	13 (25.0)	
Fusiform	75 (70.1)	36 (65.5)	39 (75.0)	
Maximum dimeter (mean ± SD)	11.8 ± 7.1	11.8 ± 7.2	11.8 ± 7.0	0.981
Size (*n*, %)				0.499
Small (< 5 mm)	4 (3.7)	1 (1.8)	3 (5.8)	
Medium (5–10 mm)	53 (49.5)	29 (52.7)	24 (46.2)	
Large/giant (> 10 mm)	50 (46.7)	25 (45.5)	25 (48.1)	

*PED* Pipeline embolization device, *TFD* Tubridge flow diverter, *SD* Standard deviation

Of the 107 PCAs, 83 (77.6%) were located at vertebral artery, 16 (15.0%) at basilar artery, 6 (5.6%) at vertebrobasilar junction, 1 (0.9%) at posterior inferior cerebellar artery, and 1 (0.9%) at posterior cerebral artery. Aneurysm morphology was fusiform in 75 (70.1%) patients and saccular in 32 (29.9%) patients. Maximum diameter of aneurysm ranges from 4 to 53 mm (mean ± *SD* 11.8 ± 7.1 mm). Four (3.7%) aneurysms are small (≤ 5 mm), 53 (49.5%) are medium (5–10 mm), and 50 (46.7%) are large or giant (> 10 mm) in size. Of the 107 aneurysms, 3 (2.8%) had undergone previous endovascular treatment (stent-assisted coiling). There is no statistical difference in demographics and aneurysm characteristics between PED and TFD patients (Table 1).

A total of 115 FDs were used for 107 aneurysms. All FDs were successfully implanted with a technical success rate of 100%. The PED was used in 55 (51.4%) and TFD in 52 (48.6%) patients. One-hundred patients used one FD, 6 patients used 2, and 1 patient used 3. Ninety (84.1%) aneurysms were treated with FD along, and 17 (15.9%) were treated with FD and adjunctive coils (Table 2).

**Table 2:** Treatment details and clinical outcomes of patients

	Total *n* = 107	PED *n* = 55	TFD *n* = 52	*P*-value
Treatment strategy (*n*, %)				0.504
FD along	90 (84.1)	45 (81.8)	45 (86.5)	
FD and coils	17 (15.9)	10 (18.2)	7 (13.5)	
Number of FD (*n*, %)				0.753
1	100 (93.5)	51 (92.7)	49 (94.2)	
≥ 2	7 (6.5)	4 (7.3)	3 (5.8)	
Complication (*n*, %)				0.737
Hemorrhagic	0 (0.0)	0 (0.0)	0 (0.0)	
Ischemic	9 (8.4)	4 (7.3)	5 (9.6)	
Angiographic follow-up (*n*, %)				0.241
Complete occlusion	65 (80.2)	35 (85.4)	30 (75.0)	
Incomplete occlusion	16 (19.8)	6 (14.6)	10 (25.0)	
Clinical follow-up time (month, mean ± SD)	25 ± 10.3	23.8 ± 12.5	26.4 ± 7.1	0.196
Clinical outcome (*n*, %)				0.954
Favorable outcome	103 (96.3)	53 (96.4)	50 (96.2)	
Unfavorable outcome	4 (3.7)	2 (3.6)	2 (3.8)	

*FD* Flow diverter, *PED* Pipeline embolization device, *TFD* Tubridge flow diverter, *SD* Standard deviation

Of the 107 procedures, 9 (8.4%) procedural-related symptomatic complications happened, and all of them are ischemia. There was no hemorrhagic complication. Three patients (2.8%) experienced transient ischemic symptoms (one is unilateral limb numbness, one is unilateral limb numbness and diplopia, one is cortical blindness), and CT or MRI revealed no infarction. They are totally recovered at discharge after medication using anti-vasospasm therapy and tirofiban intravenously, etc. Four patients (3.7%) suffered mild or severe neurologic deficit including hemiparesis, dysarthria, dysphagia, and/or vertigo, and CT/MRI confirmed brain stem or cerebellum infarction. Intra-stent thrombosis or perforator occlusion is potential mechanisms. Tirofiban, volume expansion, anti-vasospasm treatment and agent-promoting collateral circulation, etc. were adopted. Physical rehabilitation treatment was recommended at discharge. Mass effect happened in 2 patients (1.9%). One presented as loss of consciousness, dysarthria, and right limb weakness 1 day after procedure and gradually recovered after medical treatment using mannitol and glucocorticoid, etc. The other one died of sudden cardiopulmonary arrest 1 day after procedure, followed by loss of consciousness and coma. Both of the two patients harbor giant aneurysms with a maximum diameter of 26 mm and 53 mm, respectively. No statistical difference is observed in terms of complications between PED and TFD groups in univariate and multivariate analysis (9.6% versus 7.3%, *p* = 0.737) (Table 2).

**Fig. 1:**
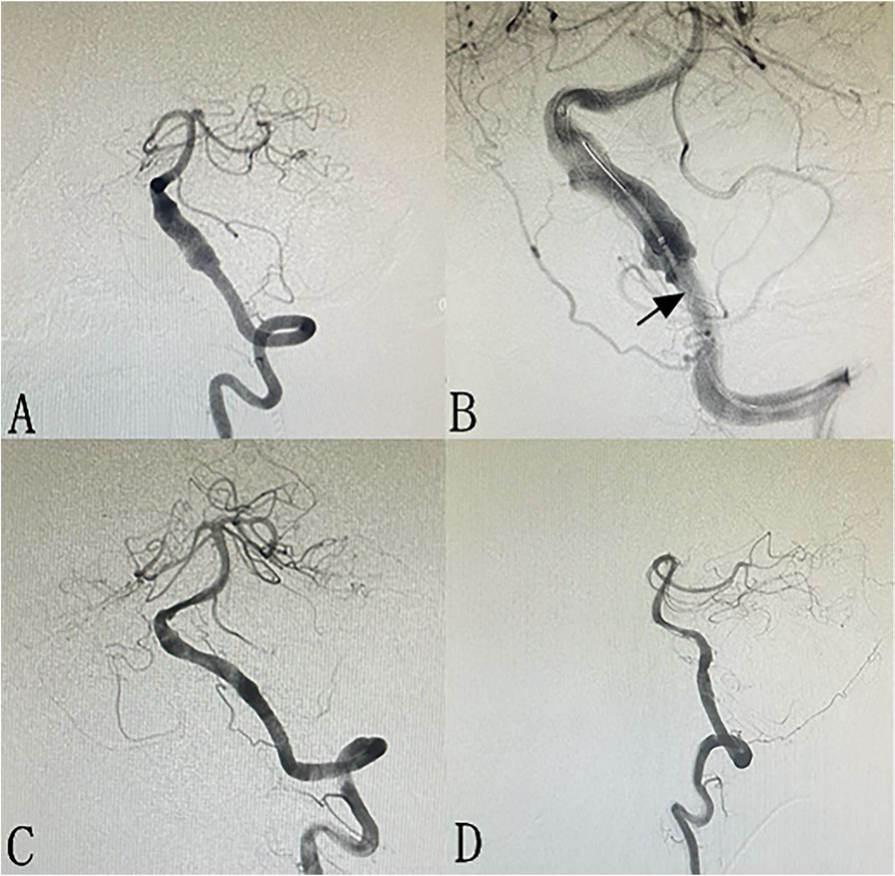
A 61-year-old male with unruptured fusiform aneurysm located at left vertebral artery (**A**). TFD is implanted for the aneurysm (**B**, arrow). Nine-month follow-up angiography showed complete occlusion of the aneurysm (**C** and **D**)

**Fig. 2:**
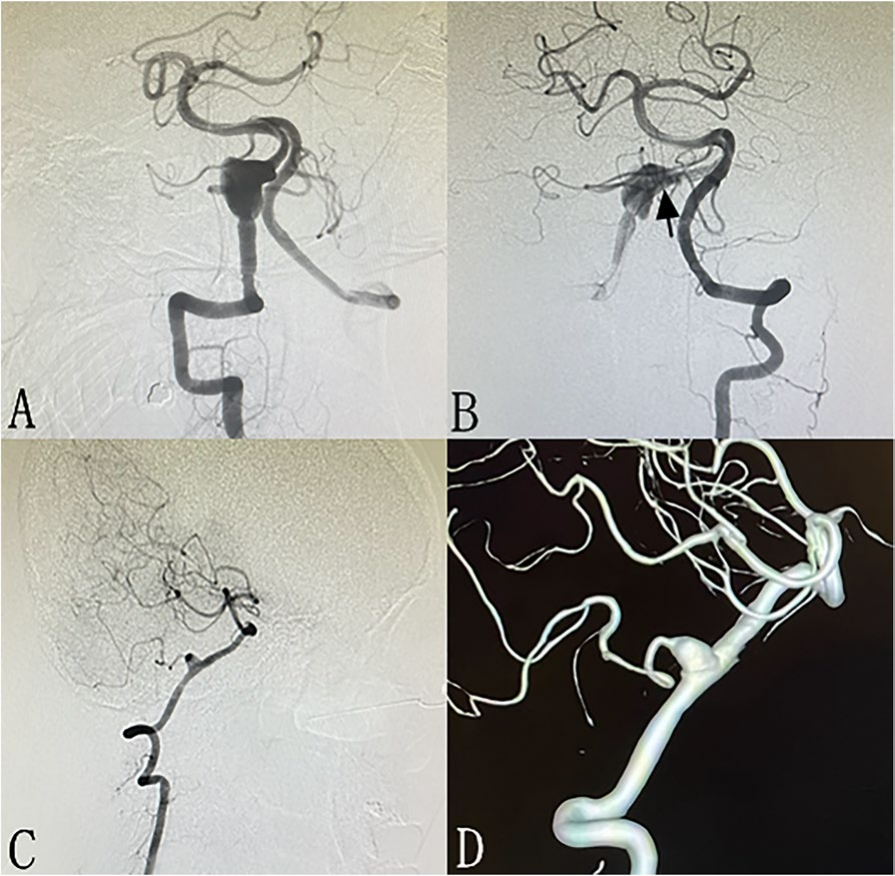
A 42-year-old female with a unruptured aneurysm located at right cerebral artery(**A**). PED (**B**, arrow) is implanted for the aneurysm, and immediate postoperative angiography showed laminar flow (**B**). Ten-month follow-up angiography showed near completely occlusion of the aneurysm with PICA patent (**C** and **D**)

A total of 81 (75.7%) patients received angiographic follow-up, and an average angiographic follow-up of 9.4 months (6–24 months) was performed. Sixty-one (75.3%) patients were followed-up with DSA, 20 (24.7%) with MRA or CTA. The overall occlusion rate is 80.2% (65 in 81 patients). Illustrate cases are shown in Figs. 1 and 2. The result revealed that complete occlusion was achieved in 35 (85.4%) patients in PED patients and 30 (75.0%) in TFD patients. In one patient, the parent artery (left vertebral artery) was occluded 9 months after TFD implantation, while the patient has no symptom due to adequate collateral circulation. The occlusion rate of PED patients is higher than that of TFD patients (85.4% versus 75.0%), while there is no statistical difference (*p* = 0.241). An average of 25-month clinical follow-up showed that the mRS at last follow-up is 0 in 98 (91.5%) patients, 1 in 3 patients, 2 in 2 patients, 3 in 2 patients, 4 in 1 patient, and 6 in 1 patient. Favorable clinical outcome was achieved in 53 (96.4%) patients in PED group and 50 (96.2%) in TFD group, respectively. The overall rate of favorable clinical outcome is 96.3%. The overmorbidity and mortality are 2.8% (3 of 107) and 0.9% (1 of 107) respectively. There is no statistical difference in terms of favorable clinical outcome rate between groups (96.3% versus 96.4%, *p* = 0.954) (Table 2).

## Discussion

The PED is the first FD for intracranial aneurysm treatment. It was approved by Food and Drug Administration (FDA) of the USA in 2011. The initial indication is large and giant aneurysms in the internal carotid artery extending from the petrous to the superior hypophyseal segments, which is later extended to small and medium aneurysms. However, the use of PED for PCAs is still off-label even though it has been proved safe and effective [11–14]. The TFD is a relatively new FD approved by FDA of China in 2018. It is a braided, self-expanding device with flared ends, which has various features that seem to predetermine its use in the posterior circulation [15]. The indication includes both anterior and posterior circulation intracranial aneurysms. In the current study, we conducted a retrospective analysis comparing the safety and efficacy between PED and TFD for unruptured PCAs. A total of 115 FD was successfully implanted for 107 PCAs. Total obliteration rate of PED and TFD was 85.4% and 75.0% at last follow-up, respectively. The occlusion rate of PED is slightly higher than that of TFD, while there is no statistical difference. Complication happened in 7.3% of PED group and 9.6% of TFD group. The rate of favorable clinical outcome at last follow-up was 96.3% (53/55) in the PED group and 96.2% (50/52) in the TFD group. The difference of complication rate and clinical outcome was not significant (*p* > 0.05). These results indicate that the safety and efficacy of the two FDs in the treatment of PCAs did not differ. Both PED and TFD are suitable for the treatment of unruptured PCAs.

Despite the above similar clinical safety and efficacy, PED and TFD have different textures and characteristics; thus, options should be made for some specific aneurysms with different characteristics. Liu et al. summarized the features of PED and TFD. The PED is made of 75% cobalt–chromium and 25% platinum, and TFD is made of nickel–titanium alloy. The application of a nickel–titanium alloy allows for improved shape-holding memory and super-elasticity. The platinum–iridium material used for the radiopaque microfilaments improves visualization of the stent during deployment [15, 16]. The size of PED ranges from 2.5 to 5.0 mm in diameter and 10 to 35 mm in length. The size of TFDs ranges from 2.5 to 6.5 mm in diameter and 12 to 45 mm in length; thus, TFD could be applied in treating aneurysms with neck or parent artery larger in diameter [17]. TFD was relatively soft with weaker radial support force and could be easily affected or displaced by movement of micro-guide wires or microcatheters in tension [18]. The PED was slightly stiffer and difficult to open, especially in the curved part of the vessel. Sometimes, repeated pushing and pulling are necessary to ensure complete opening. Compared with PED, TFD have a relatively higher shortening rate due to slightly higher vascular adaptation. Both the PED and TFD may be more suitable for cases with more uniform vessel diameters, while the TFD with a slightly larger diameter may be more suitable for aneurysmal arteries with large distal and proximal lumen disparities. During treatment, it is advisable to choose a slightly longer length for the TB than for the PED for the same cases [10]. The FD is the most classical FD and still mostly used by neurological interventionalists. As with the accumulation of experience, indications and clinical use of FD will continue to expand.

Flow diversion in the posterior circulation is associated with higher risks than with the anterior circulation [19, 20]. The large number of perforating and branching arteries of the posterior circulation potentially increases the risk in flow diversion, exposing patients to thromboembolic complications and brain stem stroke. The occlusion of invisible perforator may be the most common cause of ischemia [21]. A meta-analysis combined 12 studies comprising 358 PCAs treated with the PED and revealed an 18% complication rate [13]. Another meta-analysis involved 301 patients who underwent FDs treatment for PCAs and showed that good functional outcomes were reported in 66.8%, and the overall mortality was 10.6%. Multivariate logistic regression showed that younger age and fewer FDs remained significantly associated with good clinical outcome [22]. Abdel et al. assessed the efficacy and safety of FDs in the management of PCAs. A total of 659 patients and 676 PCAs were included. The pooled rates of hemorrhage, ischemia, and mortality and neurological morbidity were 2%, 8%, 7%, and 6%, respectively. Regression analysis showed that elderly patients and females had higher morbidity [23]. Our findings reveal complications in 8.4% and good outcomes in 96.3%. The overall morbidity and mortality are 2.8% and 0.9%, respectively. In the present cohort, the rate of procedural complications and mortality was lower than reported literatures. This may due to high percentage (77.7%) of aneurysms located at vertebral artery, which contains less perforators and relatively safe to use FD. Several strategies may also have contributed to low complications rate. The antiplatelet therapy is guaranteed to be adequate, and platelet inhibition rate and CYP2C19 gene-type testing are necessary. Candidate therapy should be used in case of clopidogrel resistance to ensure platelet inhibition rate. Experience for the FD was another factor, and with increasing experience, the technique-related complications would certainly be decreased. After the FD had been deployed, it was sometimes useful to “massage” with a micro-guide wire or a microcatheter to prevent possible stent displacement. The anchoring distance of the FD should be sufficient, and a sufficient anchoring distance could prevent the stent to shorten, especially in tortuous or curved arteries. Application of small doses of tirofiban during and 24 h after the procedure is also helpful. The use of tirofiban will not increase hemorrhagic risk but can significantly decrease ischemic complication risks [24].

The occlusion rate of FD for PCA differs in various studies. Zhou et al. conducted a single-center cohort study of 28 patients with intracranial aneurysm treated with TB [17]. A mean follow-up period of 9.9 months showed that complete occlusion rate was 72%. A meta-analysis assessed the efficacy of FDs in the management of PCAs. Fourteen studies with a total of 676 posterior circulation aneurysms were included. Complete occlusion occurred in 82.4% of the PCA. Posterior circulation aneurysms can be effectively treated with FDs [23]. A literature review looking at treatment of basilar artery aneurysms with FDs reported complete/near-complete occlusion rates, ranging from 58.3 to 87% (mean, 75%) [25]. These findings are consistent with our results with an occlusion rate of 80.2%. The mean follow-up time of this study was 10.3 months on average. Long-term follow-up would show higher occlusion. Previous literatures have summarized a variety of predictors of occlusion. A meta-analysis involved 84 articles reporting FDs for the treatment of PCAs that complete aneurysm occlusion was 65.1% with a mean angiographic follow-up time of 11.3 months. Multivariate logistic regression found that age and size remained significant predictors of angiographic occlusion, with older age and giant aneurysms associated with decreased aneurysm occlusion [22]. A single-center experience also showed that age and aneurysm size are predictors of occlusion in BA aneurysms. Older in age or large in size correlated with aneurysm persistence for posterior circulation aneurysms [26]. The use of adjunctive coils has been associated with increased occlusion rates [27]. Foreman et al. concluded that aneurysms harboring large amounts of pre-treatment thrombus were associated with lower rates of complete occlusion [28]. The effect of flow diversion is closely related to correct selection of the flow diverter. The selection of appropriate diameter of the stent is very important. The closer the stent diameter is to the true diameter of the vessel, the easier it is to open and the better it will adhere to the wall [18]. Factors related with occlusion need to be confirmed by large cohorts.

### Limitations

Some limitations existed in this study. The sample size is relatively small. The retrospective and single-center study design will affect the generalization of the outcome. During the angiographic follow-up, the rate of patients followed with DSA is relatively low. The angiographic follow-up is insufficient to evaluate long-term treatment outcomes and complication rates. More patients need longer angiographic follow-up with DSA. This is not a consecutive case series because patients who meet the exclusion criteria were excluded, so there may have been a selection bias during patient sampling. Therefore, prospective multicenter studies are needed.

## Conclusions

The current study found no difference in complication, occlusion, and clinical outcome between PED and TFD for unruptured PCAs.

## Data Availability

The datasets used and/or analyzed during the current study are available from the corresponding author on reasonable request.

## Abbreviations

PCA: Posterior circulation aneurysm
FD: Flow diverter
PED: Pipeline embolization device
TFD: Tubridge flow diverter
mRS: Modified Rankin Scale
